# Effective Treatment of Basal Cell Carcinoma with a Topical Enzymatic Mixture Enriched in Bromelain: Summary of Proof-Of Concept Clinical Studies on the First 22 Tumors

**DOI:** 10.3390/jcm13216624

**Published:** 2024-11-04

**Authors:** Lior Rosenberg, Yaron Shoham, Brian Berman, Stephen K. Tyring, Michael D. Tharp, Adam J. Singer

**Affiliations:** 1 Department of Plastic and Reconstructive Surgery, Soroka University Medical Center, Ben Gurion University, P.O. Box 15000, Beer-Sheba 8443944, Israel; yshoham@bgu.ac.il; 2 Department of Dermatology and Cutaneous Surgery, University of Miami, 1600 NW 10th Ave, Miami, FL 33136, USA; bbmdphd@gmail.com; 3 Department of Dermatology, McGovern School of Medicine, University of Texas Health Science Center, Houston, TX 77401, USA; styring@ccstexas.com; 4 True Blue Clinical Research, Tampa, FL 33511, USA; mdtharp49@gmail.com; 5 Department of Emergency Medicine, Renaissance School of Medicine, Stony Brook University, HSC-L4-080, Stony Brook, NY 11794-8350, USA; adam.singer@stonybrook.edu

**Keywords:** bromelain, CPEEB, enzymatic debridement, enzymatic tumor’ therapy, BCC, SCC, solar actinic keratosis, KC, 5FU, imiquimod, PDT

## Abstract

**Background/Objectives:** Basal cell carcinoma (BCC), the most prevalent form of human cancer, is traditionally treated by surgical and alternative destructive or topical chemical means, each with its advantages, challenges, and drawbacks. We describe our experience treating BCCs with a topical concentrate of proteolytic enzymes enriched in bromelain (CPEEB) sourced from pineapple stems. CPEEB has strong proteolytic, antitumor–proapoptotic, and inflammation modulation activities, and is approved for debridement of deep burns and starting phase 3 trials for chronic wounds. **Methods:** In the first proof-of-concept (POC) study, six BCCs on three individuals were treated with five to six daily CPEEB 10% topical applications under a zinc oxide-based occlusive dressing for 9–12 h each during a period of up to 10 days. These patients were followed for up to 4 years. In an additional two POC studies, 16 patients with one BCC each were treated every other day for a total of seven applications of topical CPEEB 5% under a variety of occlusive dressings. The wounds were followed for up to 2 months before undergoing diagnostic excisional biopsy. **Results:** In the first study, clinical assessment of the BCCs and two excisional biopsies after 6 months suggested that all lesions were eradicated with spontaneous healing within ~2 weeks without clinical or histological recurrence for over 4 years. In the two subsequent studies, 16 histologically diagnosed superficial and nodular BCCs were treated using four application techniques. Excisional histology after 2 months confirmed BCC eradication in seven of the patients. In nine patients, with compromised occlusive dressings, histological eradication was incomplete. Treatment was well tolerated by all patients with the expected local skin reactions, which completely healed within 2–3 weeks. **Conclusions:** These are POC preliminary studies aimed at indicating the potential efficacy and feasibility of topical CPEEB in eradicating BCC. In these studies, topical CPEEB 10% and 5% resulted in complete eradication of the BCC when appropriately applied. CPEEB was well tolerated in all patients, and all treated sites’ erosions healed without scars in <3 weeks. Further research is necessary to corroborate the results, refine the application technique, and complete the regulatory process.

## 1. Introduction

Skin cancer is the most prevalent form of cancer in humans, with over 5.4 million new cases a year in the U.S. alone, with low-risk basal cell carcinoma (BCC), mainly superficial (SBCC) or nodular (NBCC), as well as non-melanoma skin cancer (NMSC), constituting roughly 80% of all cancers [1]. Epidermal keratinocytes are the cellular origin of BCC, squamous cell carcinoma (SCC), and other rare carcinomas, which are termed keratinocyte carcinomas (KCs), as is the common precancerous actinic (solar) keratosis (AK) lesion. Most KCs are caused by solar (actinic) ultraviolet (UV) damage to DNA. The primary approach to BCC treatment is surgical, such as excisional biopsy and Mohs micrographic surgery, alongside non-surgical destructive options like cryosurgery, laser ablation, or mechanical abrasion. Alternatively, for cases with extensive spread or for patients unwilling or unable to undergo surgery, topical chemotherapy agents like 5-fluorouracil cream and imiquimod or photodynamic therapy (PDT) are commonly used [2,3,4,5,6,7,8,9,10].

Surgery and destructive techniques, despite their availability, require specialized skills and facilities, are invasive, often result in scarring, and come with a high cost. Complications, though infrequent, can lead to local infections, wound dehiscence, prolonged healing times, and noticeable scarring. The high occurrence of actinic lesions has driven the demand for effective non-surgical treatments. Current topical medications include 5FU, which disrupts DNA synthesis, and the immunomodulator imiquimod, both of which require extended treatment periods. Photodynamic therapy is another option, targeting multiple actinic keratoses and SBCCs (but not the common NBCCs), with photosensitization treatments spanning several weeks (up to 6–12 weeks).

These therapies generally offer an 85–90% success rate in the first year, with a recurrence-free rate of 62.7% over five years for 5FU and 80% for PDT and imiquimod. These therapies that require 6–12 weeks to complete involve side effects that include local pain, redness, swelling, skin irritation, scarring, and systemic adverse events lasting for months, often causing prolongation or cessation of treatment [5,6,7,8,9,10]. Thus, there is an unmet need for a safe and effective non-surgical, rapid, non-invasive alternative in the management of low-risk skin cancer.

As previously published [11], an active pharmaceutical ingredient (API) comprising a concentrate of proteolytic enzymes enriched in bromelain (CPEEB 10%), extracted from the stems of pineapples, was used for the topical treatment of BCC. This API is already approved for eschar removal (debridement) of deep burns in the US and throughout Europe, in addition to many other parts of the world (under the tradename NexoBrid), and is in the late clinical development stage for debridement of and wound bed preparation for chronic ulcers (under the tradename EscharEx) [12,13]. In addition to its proteolytic properties, several studies have demonstrated that bromelain has anti-tumor, proapoptotic, and inflammation-modulating activities [14,15,16,17,18,19,20,21,22,23,24,25].

In a study utilizing a mouse model with chemically induced skin papilloma using dimethylbenzene(a) anthracene and 12-O-tetradecanoyl phorbol-13-acetate (DMBA–TPA), the application of bromelain on the skin was observed to reduce the number of tumors and their size. This study revealed that the mechanism behind bromelain’s tumor-suppressing effect involves the activation of p53, alterations in the Bax/Bcl-2 balance favoring apoptosis, the activation of caspase enzymes, a reduction in Cox-2 enzyme levels, and the obstruction of the NFκB signaling pathway through modulation of the MAPK and Akt/PKB pathways [17,18]. Furthermore, bromelain was found to reduce the growth rate of human epidermoid carcinoma-A431 and melanoma-A375 cell lines, as well as reduce keratinocytes’ capacity for attachment and uncontrolled proliferation [19]. Bromelain also interferes with the process of metastasis by preventing platelet aggregation and reducing the invasiveness of tumor cells in the B16F10 murine melanoma cell line [14]. The pro-apoptotic properties of bromelain were also demonstrated in oral squamous cell carcinoma cell lines Ca9-22 and SCC25 [16]. The inflammation-modulating effects may contribute to the scarless healing of the abraded/eroded treated sites and burns [13,14,15,16,17,20,23,24,25].

We hypothesize that CPEEB represents a multifaceted synergistic approach to non-melanoma skin cancer, specifically for the treatment of keratinocyte carcinomas such as BCC, SCC, and AK, exploiting the proteolytic activity to target and destroy the main tumor tissue bulk and the underlying UV-damaged basal membrane. The anticancer, pro-apoptotic effect should eliminate residual tumor cells that may remain in the treatment site. These effects result in a deeply eroded ulcer of the BCC site, including the adjacent basal membrane, with more superficial ulceration–erosion of the non-cancerous skin surrounding the tumor, combined with the specific antitumor effect. Furthermore, the excellent safety profile of CPEEB and its inflammatory modulating properties are expected to promote fast, effective, and scarless healing of the treated area.

This hypothesis has been tested in a small, published proof-of-concept (POC) study including three patients with six BCCs, demonstrating the safe and effective eradication of clinically diagnosed, superficially invasive basal cell carcinoma, nodular BCC, and one invasive morphea-type BCC (MBCC) [11].

The first POC study was followed by two additional small POC studies, all exploring various methods of applying CPEEB 5%, which are the subject of this manuscript. The primary objective of the studies was to assess the safety and tolerability of the CPEEB. The secondary objective was to assess the efficacy of the CPEEB in eradicating the BCCs in different CPEEB concentrations and application approaches. 

## 2. Materials and Methods

Study Designs: In the first published proof-of-concept study, three patients, two females and one male, aged 35, 70, and 72 years old, with three clinically diagnosed, superficially invasive basal cell carcinoma (SBCC)—two nodular BCCs and one invasive morphea-type BCC (MBCC) <10 mm—were selected on a consecutive basis and treated with 2–4 mL of topically applied 10% CPEEB 5–6 times over a period of less than 2 weeks. The treated BCC site was surrounded by a zinc oxide adhesive barrier to contain a 3–5 mm-thick layer of CPEEB 10%, and was covered by two layers of Opsite film, an occlusive transparent dressing (Smith & Nephew, Andover, MA, USA). This treatment course ended as expected with a superficial erosion of the treated sites and a deeper small crater, a little larger than the original tumors’ size, all healing without scars in 2–3 weeks. All lesions were clinicaly assessed as completely cleared, with two lesions (an SBCC and the MBCC) that were excised 6 months post treatment (Mohs technique), confirming complete histological clearence. At present, over 4 years later, none of the lesions have recurred [11].

We planned to conduct two additional prospective open-label trials to evaluate the safety and efficacy of CPEEB 5% in the treatment of BCC. Both studies included two treatment groups differing in the dosing regimens: (1) seven treatment applications applied every other day, and (2) seven daily treatment applications. The studies were conducted according to the guidelines of the Declaration of Helsinki. A similar protocol (MW2020-11-26) was approved by the local institutional review boards and informed consent was obtained from all subjects involved in the study. One (US) study conducted at 3 centers was registered at clinicaltrials.gov (NCT05157763). In addition, a parallel study with a similar protocol design was initiated in an Israeli hospital (MOH number 2021-04-21-009660 and Soroka IRB 0385-20).

**Figure 1:**
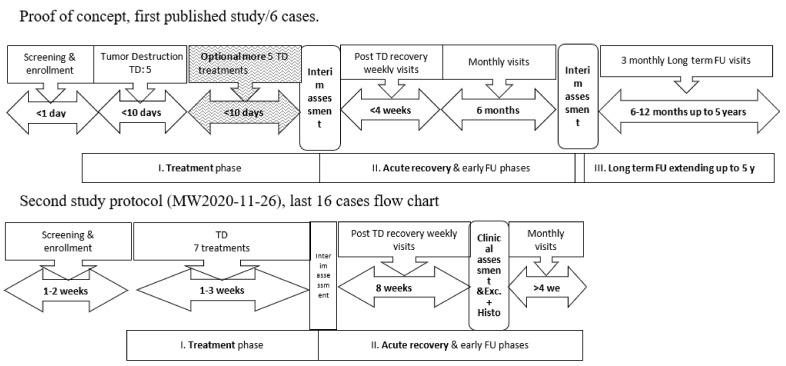
Schematic description of the study design. In the first proof-of-concept (POC) study, the enrollment (1 day) and treatment phases were shorter (no diagnostic histology and only 5–6 daily treatments) compared to the second study. The post-treatment phases were longer in the first POC study (excisional biopsy after 6 months for two lesions and follow up for >4 years) compared to the second study, where all underwent excisional biopsy after 2 months and follow-up until wound closure at 1 month. The third study, conducted in Israel, followed the same scheme as the second study in the US. The last two studies ended in wide-margin complete excisions without additional follow-up. The shaded area represents optional treatments that were only used on one of the lesions once (sixth application). TD: tumor destruction; FU: follow-up.

Setting and Subjects: In the two additional studies (protocol MW2020-11-26), patients with superficial or nodular BCC < 10 mm were selected on a consecutive basis. The patients were treated at 4 medical centers (3 in the US and 1 in Israel), with experienced dermatologists or plastic surgeons serving as the principal investigators. In these two studies, 16 adult patients (15 in the US and 1 in Israel) with one BCC each that was diagnosed clinically and confirmed by shave biopsy were enrolled and received treatment. Of the 16 patients, with ages ranging from 39 to 89 years, 13 were males. Twelve of the lesions were SBCC, with the remainder being NBCC. The studies’ designs are depicted in Figure 1.

**Figure 2:**
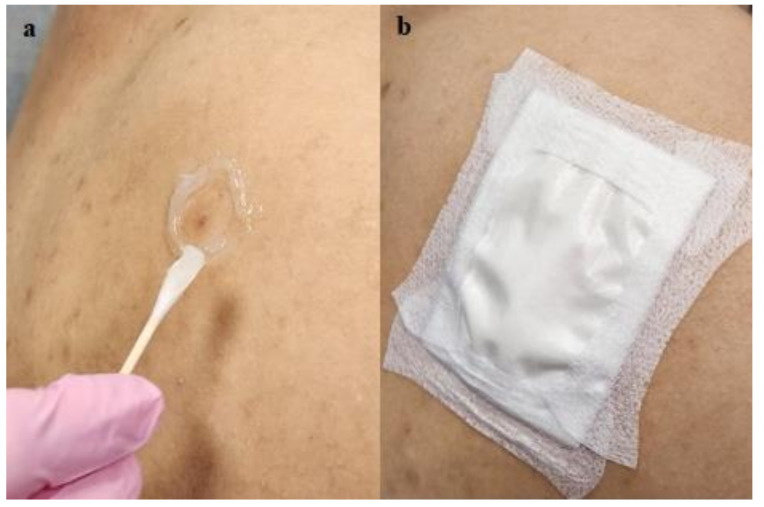
Group 1 lesion. (**a**) Application of Aquaphor adhesive barrier and (**b**) coverage by Telfa.

Interventions: In all patients, CPEEB with a concentration of 5% (4 g in 10 mL water) was applied topically under an occlusive dressing within an adhesive barrier made of Aquaphor (petrolatum ointment, Beiersdorf AG, Beiersdorfstraße 1-9, 22529 Hamburg, Germany) (Figure 2) or colostomy rings (Coloplast Brava Protective Seal) for a period of 8–12 h. Different occlusive dressings were used by the clinical sites. The volume of CPEEB applied was dependent on the volume of the occlusive chamber, which varied based on the thickness of the adhesive barrier around the treated site, the nature of the occlusive film, and the skin topography.

**Figure 3:**
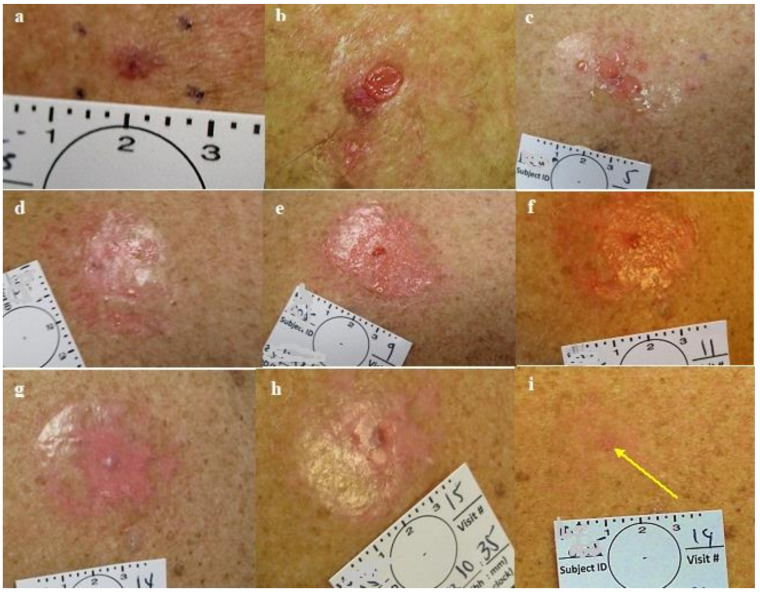
Group 1 lesion. (**a**) Nodular BCC pre-treatment, (**b**) post first treatment, (**c**) post treatment #2, (**d**) one day post treatment #3, (**e**) post treatment #4. (**f**) post treatment #5, (**g**) one day post treatment #6 and pre-treatment #7, (**h**) post treatment #7, (**i**) two months post last treatment, before excisional biopsy; see yellow arrow: clinical and histological clearing.

After the first 11 patients were treated, a data safety monitoring board (DSMB) convened, and it was decided to replace the Aquaphor adhesive barrier with a standard colostomy ring (Figure 3) in order to simplify and standardize the application procedure and prevent leakage of the topical agent. The corrective action was implemented on 4 additional patients. After treatment of these patients, an additional, last 16th patient was treated with a double colostomy ring (double-volume treatment chamber) and a double layer of an occlusive film to prevent evaporation of the CPEEB. Thus, in addition to the first published POC study [11] in these 2 studies, there were 4 treatment groups based on the method of application (Table 1). Group 1 (n = 5) was treated with Aquaphor and a Telfa (non-adherent occlusive dressing, [Covidien, Mansfield, MA, USA]). Group 2 (n = 6) was treated with Aquaphor (in one case, additional silicone gel (Scar-away gel, Alliance Pharma, Cary, NC, USA) was applied) and an Opsite Flexogrid transparent dressing (Smith & Nephew, Andover, MA, USA) and a hydrocolloid dressing (Duoderm, Convatec, Bridgewater, NJ, USA) dressing. Group 3 (n = 4) was treated with a colostomy ring + Tegaderm dressings, while Group 4 (n = 1) was treated with two colostomy rings + a double layer of Tegaderm.

The total duration of the study was approximately 14–17 weeks (see Figure 1—schematic study timeline). After completion of the treatment phase, the resulting wound was treated conservatively with a petrolatum-based ointment until wound closure. A low-potency corticosteroid ointment was applied for several days towards the end of the wound-healing phase to prevent formation of any granulation tissue at the discretion of the principal investigator. Two months after treatment, all BCC sites were excised and assessed histologically for complete clearance.

Outcomes: The safety endpoints included the incidence and severity of treatment-emergent adverse events (TEAEs) and serious TEAEs as well as the presence of abnormal vital signs and pain. The efficacy endpoints were the proportion of patients who reached complete histological clearance of their BCC at the end of the treatment period and the proportion of patients who reached complete clinical clearance of their tumor at the end of the treatment period prior to surgical removal. Additional endpoints included patient compliance with treatment and time to complete healing. Post hoc analyses evaluated efficacy endpoints in relation to the different application techniques that were implemented in these studies.

Statistical Analysis: Descriptive statistics were used to summarize the data. Categorical data were summarized with numbers and percentages. Continuous data were summarized as means and standard deviations or medians and interquartile ranges based on data distribution. No formal hypothesis testing was performed.

## 3. Results

All patients tolerated the treatments very well, reporting a tingling–pricking sensation 2–4 h after application, but none asked to discontinue the treatment.

In the 15 patients treated under protocol MW2020-11-26 (groups 1, 2, 3 from the US), 2 of 4 (50%) patients showed complete histological clearance of SBCC, and 4 of 11 patients showed complete histological clearance of nodular BCC. Clinical assessment of clearance was more favorable, with the same 50% clearance rate for SBCC but clearance in 9 of 11 (81.8%) patients with NBCC. The last, 16th patient in Israel (group 4) had an NBCC that was clinically and histologically cleared after 2 months.

**Figure 4:**
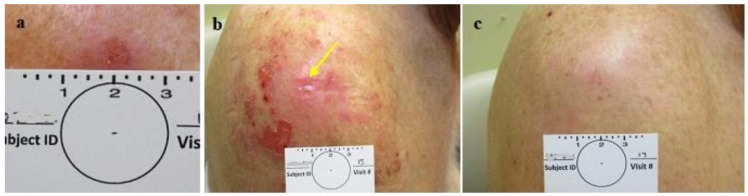
Group 2 lesions. CPEEB dripping out, underneath Aquaphor irritating the exposed skin (**a**). SBCC pre treatment, (**b**) post 7 applications (arrow points to the lesion), (**c**) 3 weeks later, complete healing, complete clinical clearence but histologically incomplete clearance.

**Figure 5:**
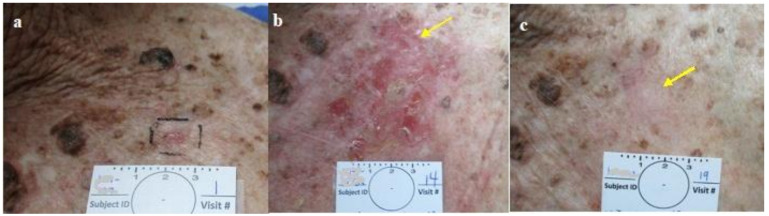
Group 2 lesions CPEEB dripping out, underneath Aquaphor irritating the exposed skin (**a**). NBCC pre treatment, (**b**) post 6 applications (arrow points to the lesion) the CPEEB irritates the skin, belowand far from the lesion, (**c**) 4 weeks later, complete healing, complete clinical clearence but histologicly suspected residual BCC.

**Figure 6:**
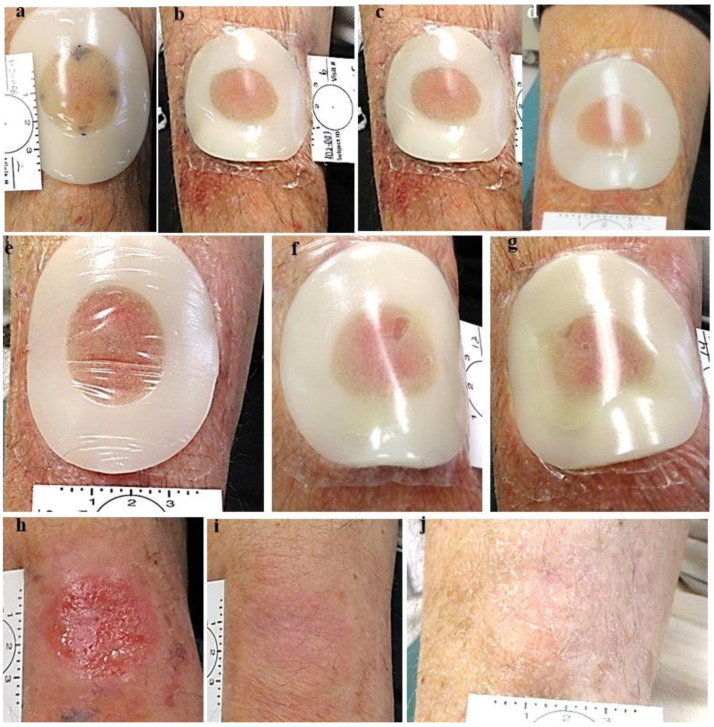
Group 3 lesions. (**a**–**d**) The colostomy ring chambers are overfilled so that, in spite of some leakage (**b**,**c**) and dehydration (**e**–**g**), enough active CPEEB would remain to successfully clinically and histologically eradicate the BCC (**h**–**j**).

**Figure 7:**
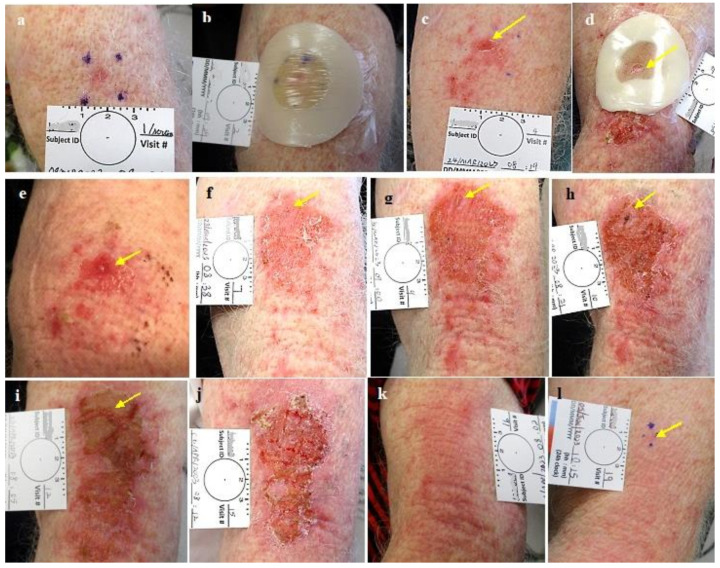
Group 3 lesions. (**a**). NBCC post shave biopsy, (**b**). #1 treatment, colostomy ring dressing applied. (**c**). Post first treatment, erosion of the lesion and its surrounding with downword leakage, (**d**). #2 treatment, leakage under a colostomy ring with the expected irritation of the exposed skin below the treated lesion, (**e**). Decreased CPEEB dosedue to leakage with decreased effect. (**f**). One day post second treatment (**g**). Post #3 treatment, marked leakage with reduced effect. (**h**). One day after #5 treatment (**i**), One day post #6 treatment (**j**). One day post #7 treatment, (**k**). Good healing at 2 weeks and (**l**). Treated area after 2 months before excisional biopsy that was positive for suspected residual BCC in a scar. The arrows point to the treated tumor.

In a post hoc critical review of the photographs documenting all the cases, we saw that the target area (skin with the BCC lesion in the center), exposed to active CPEEB 5%, was irritated or abraded after >3 applications and slightly eroded at the end of the seventh treatment (Figure 4, Figure 5, Figure 6 and Figure 7), allowing for an assessment of exposure to the CPEEB 5% and an understanding of the reasons for its failure.

The treatment failed to eradicate the lesion in cases where the volume of the treatment chamber was insufficient to allow the CPEEB to completely cover the lesion that protruded above its level or when Aquaphor dripped down onto the lesion in the treatment chamber, or when a disrupted barrier allowed CPEEB 5% to leak out or to desiccate (Table 1).

The use of Aquaphor (Figure 2) as a barrier was challenging and difficult to handle. It often liquified and then leaked after a few hours, sometimes dripping onto the treatment area possibly covering the lesion. (Figure 4, Figure 5 and Figure 6). In sites where the barrier was disrupted and the skin beyond the barrier was exposed to the leaking CPEEB, it was irritated, showing the exact pattern of the leakage and a reduced effect on the target area (Figure 4 and Figure 5). It seems that the Telfa occlusion was more effective in containing the CPEEB than the Opsite Flexogrid/Duoderm (group 1 compared to group 2). The challenging and unreliable Aquaphor adhesive barrier was the reason that a standard 2.5 mm-thick colostomy ring (Coloplast Brava Protective Seal) was chosen to replace the Aquaphor (Group 3). However, the thin ring was found to restrict the chamber’s volume, being effective only in one case when the chamber was overfilled, compensating for the thin ring and slight leak, and eradicating the NBCC (Figure 6). The ring’s application was challenging, often leaking and thus reducing the already limited volume of CPEEB (Figure 6 and Figure 7).

**Figure 8:**
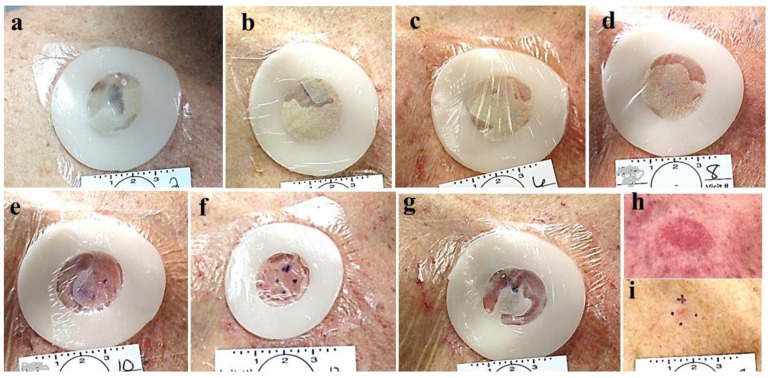
Group 3 lesions. Evaporation of the CPEEB water through the occluding film. Dry CPEEB crusts are seen on the film’s inner surface in all applications (**a**–**g**). At the end of the treatment (**g**), with a minimal, insuficient effect on the target lesion after last application (**h**) and surrounding skin, and the final outcome after 6 weeks before excisional biopsy (**i**).

In cases where the CPEEB gel’s water evaporated through the vapor-permeable, single-layer polyurethane film (Tegaderm), the enzyme was inactivated, as witnessed by the skin that was not affected (Figure 8).

In the last case (Group 4), mitigating the previous studies’ experiences, two colostomy rings, one on top of the other, forming a 5 mm-thick barrier, were carefully applied to the skin and covered with a double layer of occluding polyurethane film (Tegaderm) to ensure it was watertight, leading to a larger, completely occlusive chamber and a complete clinical and histological clearance of the NBCC in seven applications. All sites healed completely within 2–3 weeks, with good cosmetic results.

### Safety Results

Most TEAEs were mild in severity, and none were considered to be severe. Except for pruritus, no TEAE of moderate severity was reported by more than one patient. Moderate pruritus was reported by two patients. In both of these patients, the pruritus was classified as a local TEAE and was considered to be treatment related.

Local AEs included pruritus, pain, burning sensation, and also skin reactions, e.g., erythema, dermatitis, irritation, erosion, minor bleeding, and secretion/discharge. The local reactions resolved shortly after removal of the CPEEB dressing. AEs other than local AEs were reported by the investigators as not related to study treatment (e.g., distant site SCC, melanoma, lipoma, urinary tract infection). No SAEs were reported in the study. There were no treatment discontinuations due to AEs.

The post-treatment healing was uneventful and was completed in approximately 2 weeks, with minimal to no scarring. In some cases, the healed skin looked better than the surrounding untreated skin after 2 months, sometimes making it difficult to identify the treated lesion site for the diagnostic excisional biopsy (Figure 3, Figure 4, Figure 5, Figure 6 and Figure 7). In order to implement the conclusions gained with these 22 cases in a larger and more appropriately powered study, the planned study was stopped at the end of this phase without proceeding to the additional second phase of a daily application in 16 lesions.

## 4. Discussion

BCC is a slow-growing, locally invasive, epidermal-originated cutaneous tumor with a metastatic rate of <0.1%. Cutaneous SCC accounts for most of the remainder of NMSC cases, all arising from dysplastic epidermal keratinocytes that also cause the common actinic keratosis, together forming the entire spectrum of the larger group of keratinocyte carcinomas. Most of these lesions are low-risk SBCC and NBCC, and the main damage that they cause is local, invading the dermis and the surrounding skin, and later propagating to deeper structures.

The diagnosis of these cutaneous tumors is rather easy to the trained eye and may be assisted by dermatoscopy and more sophisticated means such as confocal microscopy or biopsy with histopathology. However, very often (especially in the most frequent low-risk smaller lesions), pretreatment diagnosis is limited to visual observation with or without dermatoscopy, and the success of treatment is defined by a short (1-year) and long-term (5-year) clinical assessment of the recurrency rate.

The treatment modality for these lesions should take into consideration their relatively low malignancy and slow growth but also their high incidence, often in the same individual, who frequently is aged and with other comorbidities. Choice of treatment for KC depends on risk stratification of the tumor, patient preference or suitability, and availability of local services. High-risk tumors have a greater risk of recurrence and propagation. Therefore, the best and safest choice may be a more extensive treatment: excisional surgery followed by histopathology, often followed by reconstructive procedures.

As most lesions are small and limited to one or only a few cm^2^, surgical excision under local anesthesia, followed by histopathologic assessment, is considered the gold standard. The surgical procedure, though considered limited and minor, can be rather costly and demanding for the patient. To be covered by the insurance provider, a positive pre-treatment diagnostic biopsy is required, adding to the patient’s discomfort and total costs. In recurrent, high-risk, and extensive KC, the gold standard treatment is wide-margin excisional biopsy followed by Mohs micrographic surgery (MMS), which is both time- and resource-consuming. If MMS is not available, excision with predetermined wide margins or additional radiotherapy may be considered. While Mohs surgery is an effective and accurate therapy, it is unfortunately not readily available, is costly, and may involve additional psychological trauma to the patients [7,9,26]. Excisional surgery always results in scars that are at least three times longer than the lesions’ diameter and carries the risk of surgical complications such as wound infection, dehiscence, delayed healing, and even a larger scar [4,7,9].

Thus, excisional biopsy is often replaced by physically destructive means such as freezing by cryotherapy or cautery by electrotherapy desiccation, where it is impossible to have a post-treatment histopathological diagnosis, and the success of treatment is determined exclusively by recurrence rate.

The large and growing incidence of these tumors and the challenges related to the available surgical/physically destructive treatments, as well as the reluctance of many patients to undergo this ordeal every few months, spurred the development and use of several non-surgical, topically destructive chemical agents. These can be divided into chemical (Aldara-Imiquimod (5%) and 5FU (5%)) and physical light therapy (photodynamic therapy). Topical therapies are generally indicated for more superficial small-size SBCC/SK, multiple lesions, advanced age, contraindication for surgery, immunosuppressed patients, patients with surgery phobia or cosmetic concerns, and due to their lower cost [5,6,7,8,9,10,11]. Topical 5FU, which inhibits DNA synthesis, is applied twice a day for up to 12 weeks, while the immunomodulator imiquimod is applied five times a week for up to 6–12 weeks. PDT based on photosensitization (2–6 weeks) is indicated for multiple solar keratosis SBCC. All may induce early and late side effects, often in association with each other, from light sensitivity, pain, edema, burns, pigmentation, and scarring, to carcinogenesis correlated to local and/or systemic immunosuppression or to the selection of PDT-resistant SCC.

All these non-surgical means are indicated for SBCC and AK but not for the common NBCC, and they share similar efficacy at 1 year (85–90% clearance), with 5-year recurrence rates of 60–80%.

While 5FU and imiquimod are not expensive, their use involves long and demanding treatment courses (6–12 weeks), frequent local complications (e.g., pain, redness, ulcerations, etc.) lasting for several months, as well as systemic AEs that deter patients from using them [8,26]. PDT treatment is more expensive and requires adequate setup and monitoring, as the entire treatment course involves a local reaction, redness, pain, and photosensitivity that may last for months [5,6,7,8,9]. As mentioned, these treatments are not indicated for the common NBCC.

Despite the availability of non-surgical treatments for many of these tumors, due to the extended treatment time and the noticeable discomfort/complications involved, their use does not adequately address the challenge of this large and growing population. There is an unmet need for safe, effective, and fast-acting non-surgical treatment modalities for all these common but demanding skin tumors. The need is for a treatment that will eradicate KC (mainly BCC, SBCC, and the more frequent NBCC) with only a few topical applications that will be readily tolerated by patients without all the sequelae of the existing methods, including scarring, and a 5-year recurrence-free rate of >90%.

CPEEB is known for its strong, multi-target proteolytic activity; its potential anti-tumorigenic/proapoptotic activity; as well as its favorable safety profile. The results of our preliminary studies point to its efficacy in eradicating SBCC and NBCC safely and tolerably when properly applied. The concentration may play a role, as in high (10%) concentration it seems to be less sensitive to the application technique than is seen with the 5% concentration [1]. The series of patients is too small and the variables too large to draw an accurate conclusion.

The proposed mode of action (MOA) of CPEEB in eradicating SBCC as well as NBCC is based on the following two activities:

1. Proteolysis of the different cutaneous structures according to their extracellular matrix (ECM) density. This strong activity quickly destroys the tumors’ matrices that differ from the normal, denser, and more resistant dermal collagenous extracellular matrix (ECM). This potent proteolysis explains the rapid (few days) eradication of these tumors, which does not depend on the long anti-tumorigenic/proapoptotic activities that are related to the tumors’ cell growth cycle, which has a much slower effect. This proteolysis, as seen in these studies, seems to be dose/application related, also affecting the denser surrounding dermis but in a much weaker way, causing only a superficial erosion of the epidermis and the basal membrane complex underneath. This superficial erosion destroys the tumor’s horizontal edges as well as the vertical edges that penetrate the basal membrane. The immediate destruction of the tumor with its adjacent edges and the basal membrane is similar to surgical excision that removes the tumor with its surrounding tissues.

The role of the basal membrane as a factor and a barrier to tumor formation and propagation is well known, as described recently by Riihilä et al. and Kavasi RM. et al. [27,28]. In syndromes where the basal membrane is genetically defective, as in epidermolysis bullosa, or when the basal membrane is damaged by UV exposure, one of the outcomes is the development and propagation of KCs such as BCC and SCC. This pathogenesis explains the role of the UV-damaged pathological matrix (basal membrane, ECM, MMPs, etc.), which allows the local UV-modified damaged keratocyte cells to coalesce, form, and spread as a KC tumor. Removing the tumor and its pathogenetic surroundings eradicates the tumor and decreases its potential recurrence, not unlike wide surgical excision of the tumor and its surrounding tissues.

2. The anti-tumorigenic/proapoptotic effect of the CPEEB, which destroys stray tumor cells that were not removed by the proteolytic activities, may persist at the treatment site [15,16,17,18,19,20,21,22,23,24].

Thus, CPEEB presents a new, topical, non-surgical means that combines an enzymatic “surgery-like” eradication of the tumor with its pathogenetic surroundings and a “chemotherapeutic” effect that is faster, safer, and better tolerated than the present 5FU, imiquimod, or PDT. CPEEB may represent a “next-generation” alternative to the present non-surgical modalities. The final outcome of fast and scarless healing of the superficial erosion is due to the preserved dermis, which is prone to epithelializing, and possibly to bromelain’s anti-inflammatory properties that modulate rapid, minimally scarred healing, as found in other studies.

In the presented preliminary studies, we encountered several challenges that should be considered in future studies:

1. With only 22 total tumors treated across multiple studies, the sample size was very small, limiting statistical power and generalizability. Thus, more rigorous clinical trials are needed to establish efficacy and safety.
2. There was no control or comparison group, making it difficult to assess relative efficacy, though the outcome of the existing treatments of such BCCs is quite well known and can be used as a historical control.
3. The patients operated on (all with lesions that were excised with healthy margins) were only followed up with for 2 months post-treatment, which is insufficient to assess long-term recurrence rates.
4. The aim of testing different application methods and timelines in small groups could demonstrate only gross flaws of each technique.
5. The efficacy endpoint of complete tumor eradication did not assess the different stages of the tumor destruction process, which could be a topic of different in-vitro or in-vivo studies.
6. The study focused mainly on superficial and nodular basal cell carcinomas, which are the most common. Thus, efficacy for other types is unknown.
7. The reduced concentration from 10% to 5% compared to the first POC may have caused the efficacy to be more sensitive to application technique.
8. The application technique should be fine-tuned following the present study’s conclusions.
9. Though histopathological diagnosis is considered the gold standard for tumor diagnosis, in clinical practice, the majority of low-risk NMSC KC (BCC, SCC, and SK) are clinically visually diagnosed, with or without dermatoscopy. Based on this non-invasive diagnosis, patients are treated (surgically and/or non-surgically) and followed up for possible recurrence. Pre-treatment histopathology is reserved for the more invasive cases and as a prerequisite for insurance coverage of the entire surgical treatment process, which involves two procedures (pre- and post-treatment surgery) and histopathology. Using biopsies and diagnostic histopathology in trials of low-risk lesions does confirm the nature of the lesion that is treated and the completness of eradication but adds a layer of costs, complexity, and scarring. The patient with small, low-risk BCC that is aware of the non-surgical, conservative alternatives may be reluctant to join such a study that involves two surgical procedures and more scarring. Thus, designing a surgery-free trial that is based on pre- and post-treatment clinical/dermatoscopy diagnosis is challenging and will involve more prolonged follow-up (up to 5 years) but may be more appealing to patients.
10. The final appearance of the treated lesions after 2 months is typical of a fresh, healing wound that easily can be diagnosed as a persistant superficial lesion. Histologically, inflammation with atypical cells (which are present at 2 months after treatment) may challenge accurate diagnosis. Thus, the timing for the final, post-treatment clearance assessment and biopsy should be carried out at the end of all healing and inflammatory processes (after 3–6 months).
11. Following healing of the CPEEB-treated sites, the skin looked intact and scarless, sometimes making the identication of the treated tumor’s exact location for assessment and diagnostic excision challenging. Thus, the tumor and its surroundings should be meticulously mapped initially on enrollment or even temporarily tatooed.

Strengths of Study: While small, a strength of the study is the inclusion of two concentrations, different application techniques, multiple clinical sites and investigators increasing its generalizability. The experienced PIs provided valuable insight regarding the challenges and advantages of CPEEB. All investigators considered the CPEEB 5% treatment to be safe, with the resulting brief, local irritation and erosion to be minimal and well tolerated by the patients, while noting its fast, complete, and scarless healing. The investigators also agreed on the necessity of establishing an easy and practical application technique and confirming the long-term recurrence rate in additional clinical trials.

Study Limitations: The main limitation of the study is the small sample size limiting the ability to explore additional application techniques, and the different treatment groups did not allow for pooling of the data in order to draw statistically significant results. The study was also not powered to demonstrate treatment efficacy or safety. Furthermore, the study also lacked a relevant control group for comparison. The study focused on superficial and nodular BCC. Therefore, efficacy for other types of skin cancers remains unknown. Multiple application techniques were used, making it difficult to draw any conclusions regarding the relative efficacy of the various application methods though the shortcoming were quite obvious. As these were proof-of-concept studies, more rigorous clinical trials are needed to establish efficacy and safety.

## 5. Conclusions

The results of these preliminary, proof-of-concept studies suggest that CPEEB may be a safe and effective means to treat SBCC and NBCC when properly appplied in an adequate dose. It may offer a rapid, safe, and dose-dependent effective next-generation treatment alternative to current therapies for BCC. Further research and larger-scale studies will be necessary to fully comprehend, demonstrate, and regulate the potential of CPEEB in the management of low-risk BCC and possibly other KC skin tumors.

## Figures and Tables

**Table 1:** Efficacy of CPEEB: relation to barrier and occlusive film.

Group %	Barrier	Occlusive Film	Watertight	Leaking Dressing	BCCs S/N/M	Clinical Clearance	Histologic Clearance
POC 10%	Thick ZnOx_2_	x2 Opsite	Yes	No	6 3/2/1	Yes, all	Yes 6/6
(1) 5%	Thick Aquaphor	Telfa+ Hypafix	Yes	No	5 2/3	Yes, all	Yes 5/5
(2) 5%	Thin Aquaphor	Flexigrid+ DuoDerm	No	Yes	6 1/5	Yes 3, No 3	No 6/6
(3) 5%	1 ring	Tegaderm	No	Yes	4 1/3	Yes 1, No 3	Yes 1, No 3
(4) 5%	2 rings	x2 Tegaderm	Yes	No	1 0/1	Yes	Yes 1/1

The 5 treatment groups of the 3 studies describing the differences in concentration (10% vs. 5%); the adhesive barriers, from zinc oxide to thick Aquaphor, thin Aquaphor, a single colostomy ring, and a double colostomy ring with occlusive films, some of which were watertight and others that allowed vapor passage but could be used in a double layer that occluded the treatment chamber. The volmes that were >2 cc, covering the lesion entirely, succeded in destroying the lesions, but when the volumes were reduced due to the disruption of the barrier and leakage of the CPEEB, small treatment chamber, or water evaporation, the lesions were not totally eradicated. In all studies, there were 22 BCCs, with 6 SBCC, 6 NBCC, and 1MBCC eradicated, and 2 SBCC and 8 NBCC that failed. In the last 2 studies 3 SBCC and 4 NBCC were eradicated. S = superficial BCC; N = nodular BCC, M = Morphea BCC.

## Data Availability

Data are contained within the article, no additional data is avaialble to the readers.

## Abbreviations

5FU	5-fluorouracil
AE	Adverse event
AK	Actinic (solar) keratosis
API	Active pharmaceutical ingredient
BCC	Basal cell carcinoma
CPEEB	Concentrate of proteolytic enzymes enriched in bromelain
DSMB	Data safety monitoring board
IRB	Institutional review board
KC	Keratocyte carcinoma
MBCC	Morphea-type BCC
MOH	Ministry of Health
MOHS	Micrographic surgery (developed by Fredric E. Mohs 1938)
NBCC	Nodular BCC
NMSC	Non-melanoma skin cancer
PDT	Photodynamic therapy
POC	Proof of concept
SAE	Serious adverse event
SBCC	Superficial BCC
SCC	Squamous cell carcinoma
TEAE	Treatment-emergent adverse events
UV	Ultraviolet
